# Supplementary material for: Antagonism in Orthotospoviruses Is Reflected in Plant Small RNA Profile
Source: Viruses. 2025 May 30;17(6):789. doi: 10.3390/v17060789 (PMC12197435; doi:10.3390/v17060789)
Supplement: Supplementary file 1 [file viruses-17-00789-s001.zip › Supplementary figures.pdf]

Supplementary figures:

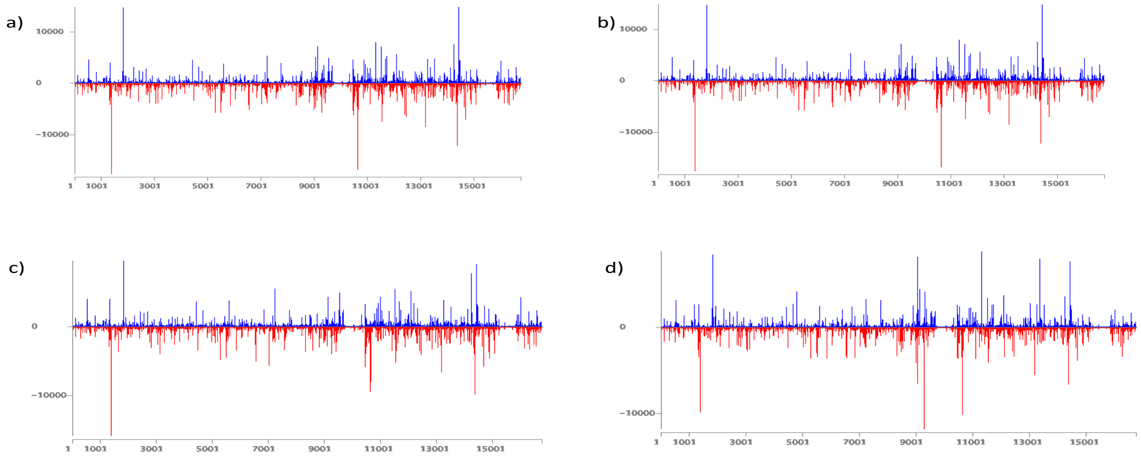

Figure S1. Hotspot distributions on the INSV viral genome. Peaks represent multiple reads aligned to the genome in the same position. Y-axis: number of reads. X-axis: nucleotide position on the viral genome. (a) INSV vsRNAs from INSV single infection replicate 1, (b) INSV vsRNAs from INSV single infection replicate 2, (c) INSV vsRNAs from Mixed infection replicate 1, (d) INSV vsRNAs from Mixed infection replicate 2. Blue: viral sense; Red: viral antisense. Figures were generated using MISIS. L segment: 1-8774nt, M segment: 8775-13751nt, and S segment: 13752- 16761nt.

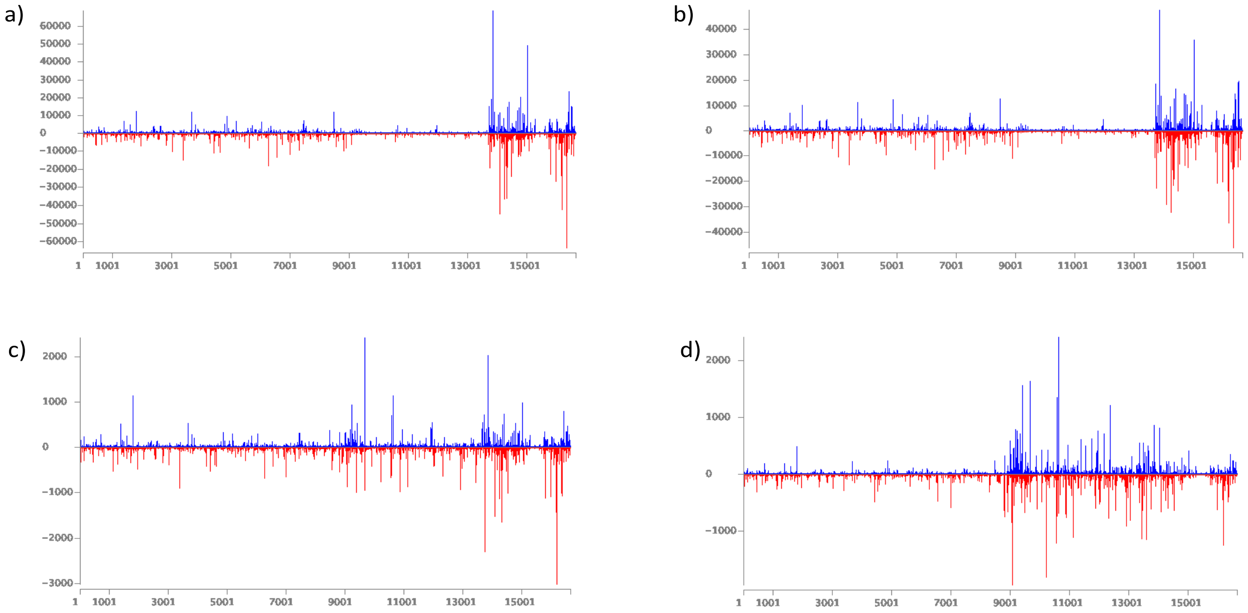

Figure S2. Hotspot distributions on the TSWV viral genome. Peaks represent multiple reads aligned to the genome in the same position. Y-axis: number of reads. X-axis: nucleotide position on the viral genome. (a) TSWV vsRNAs from TSWV single infection replicate 1, (b) TSWV vsRNAs from TSWV single infection replicate 2, (c) TSWV vsRNAs from Mixed infection replicate 1, (d) TSWV vsRNAs from Mixed infection replicate 2. Blue: viral sense; Red: viral antisense. Figures were generated using MISIS. L segment: 1-8914nt, M segment: 8915-13679 nt, and S segment: 13689- 16663nt.

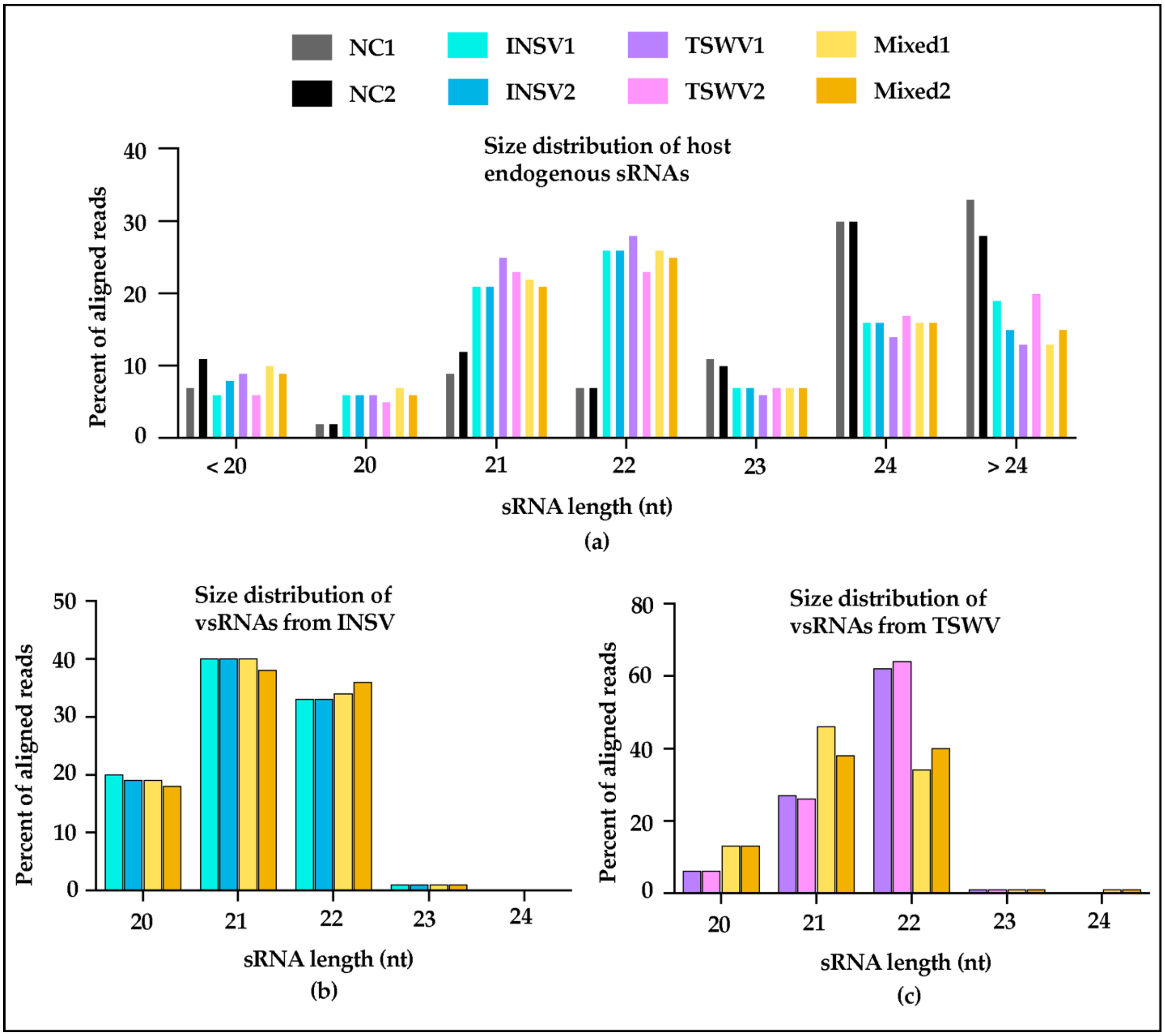

Figure S3. Summary of small RNA reads based on their size. NC: mock-inoculated negative control. Size distribution of host endogenous sRNAs form all the samples (a), INSV vsRNAs from both replicates of INSV single and mixed infection (b), and TSWV vsRNAs from both replicates of TSWV single and mixed infection (c).
